# Personality traits and workplace bullying among contract trainee doctors in Malaysia

**DOI:** 10.1016/j.heliyon.2023.e23625

**Published:** 2023-12-12

**Authors:** Halim Ismail, Dzualkamal Dawam, Nor Azila Muhd Aris, Sheng Qian Yew, Hanis Ahmad, Chan Chee Hoong David, Mohd Hafiz Baharudin, Zhe Shen Huam, Hibatul Hakimi Jamaludin

**Affiliations:** Department of Public Health Medicine, Faculty of Medicine, Universiti Kebangsaan Malaysia Medical Center, Kuala Lumpur, Malaysia

**Keywords:** Workplace bullying, Contract trainee doctors, Personality traits

## Abstract

**Introduction:**

Workplace bullying (WPB) among trainee doctors is a concerning problem in Malaysia. However, there is still limited understanding regarding the influence of trainee doctors’ personality traits on WPB. Furthermore, the impact of contract employment status on WPB among trainee doctors is not yet well-defined. To address these gaps, this study was aimed to determine the prevalence of WPB among trainee doctors and to examine the association of sociodemographic characteristics, job characteristics, and personality traits with WPB among trainee doctors in Malaysia.

**Methods:**

A multi-center cross-sectional study was conducted with 264 trainee doctors in Selangor, Malaysia. Eligible participants were provided with sociodemographic characteristics questionnaire, job characteristics questionnaire, WPB questionnaire, and the Big Five Inventory-10 (BFI-10). Chi-square tests were used to examine the association between: (i) sociodemographic characteristics and WPB, (ii) job characteristics and WPB; and (iii) personality traits and WPB. Multivariate logistic regression was performed to evaluate the association between the significant independent variables (as determined from Chi-square tests) and WPB.

**Results:**

The prevalence of WPB was 45.1 %, with verbal abuse being the most common form of bullying (46.2 %). Chi-square test showed that only marital status and low agreeableness were significantly associated with WPB. Subsequently, multiple logistic regression demonstrated that being married (OR: 1.866; 95 % CI: 1.077–3.234) and low agreeableness (OR: 2.287; 95 % CI: 1.169–4.473) were significant predictors of WPB.

**Conclusion:**

The high prevalence of WPB among trainee doctors could be attributed by marriage and low agreeableness personality traits in this population. In order to minimise WPB and maximise workforce potential, it is essential for healthcare institutions and medical training programmes to recognise this vulnerabilities and take steps to protect and support trainee doctors who are married and/or with low agreeableness personality trait.

## 1 Introduction

Workplace bullying (WPB) refers to the repetitive occurrence of disrespectful, abusive, inappropriate, threatening, or intimidating behaviour that is directed towards an individual or a group of individuals [1]. It may involve emotional or physical harm, disruption, insults, and is often motivated by a perceived or actual power imbalance. The aim of WPB is typically to exert control, undermine, embarrass, threaten, or cause harm to the targeted individuals [2]. WPB can be observed in different industries, including the health care sector [3]. Its repercussions extend beyond individual experiences, impacting the entire healthcare system at an organisational level. This includes creating a hostile work environment, undermining the organisational culture, increasing the likelihood of occupational accidents, and contributing to medical errors [4]. Additionally, WPB has the potential to adversely affect the well-being of all health care workers on an individual level. This can result in psychological distress, depression, burnout, physical health issues, and increased sick absence [5]. Ultimately, all these factors compromise the quality of care provided and contribute to negative outcomes for patients [6].

Trainee doctors (i.e., recent medical school graduates who are in the early stages of their medical careers) are particularly vulnerable to experiencing WPB due to their status as fresh graduates and working on a temporary basis in hospitals. The hierarchical structure of medical training, which often considers bullying as a necessary part of the learning process, influence WPB specifically, thereby exacerbates the situation. It is not surprising that the prevalence of WPB among trainee doctors worldwide is reported to be high, ranging from 30 % to 95 % [4]. A local study conducted in 2017 found that 13.3 % of trainee doctors experienced WPB [7]. However, this study was conducted at a time when all trainee doctors in Malaysia were still offered permanent positions. In 2017, the Ministry of Health Malaysia began providing contract positions to all medical graduates, instead of the permanent positions that were previously offered [8]. This measure was introduced to address the issue of oversupply of medical graduates in the country. Therefore, the prevalence and determinants of WPB among trainee doctors employed on a contract basis after 2017 remain unclear. It should be noted that trainee doctors on contract face reduced benefits and lack of promotion, hence, more likely to experience workplace discrimination [9]. Furthermore, while the impact of personality traits such as neuroticism, agreeableness, extraversion, conscientiousness, and openness on WPB has been extensively researched in various industries [[10], [11], [12]], the influence of these personality traits on WPB specifically among trainee doctors remains unexplored.

Given the limitations outlined above, the objectives of present study were two-fold. Firstly, we aimed to determine the prevalence of WPB among trainee doctors in Malaysia. Secondly, we sought to examine the association of sociodemographic factors, job characteristics, and personality traits with WPB among trainee doctors in Malaysia.

## 2 Methods

### 2.1 Study design

A multi-center cross-sectional study was carried out from October 2020 to March 2022 among trainee doctors employed at all training hospitals in Selangor, Malaysia.

### 2.2 Study setting

The study was planned to be conducted across all seven public tertiary hospitals in the state of Selangor, Malaysia. These hospitals are referral centers accredited by the Ministry of Health, Malaysia for training of trainee doctors. However, since Sungai Buloh Hospital, which is an infectious disease hospital, was heavily involved in treating COVID-19 positive patients at the time of study recruitment, it was not included in the present study. As a result, participants were only recruited from the remaining six hospitals in the state of Selangor, Malaysia, including Ampang Hospital, Selayang Hospital, Serdang Hospital, Kajang Hospital, Klang Hospital, and Shah Alam Hospital.

### 2.3 Study participants

Participants were invited to participate to the present study if they were: (i) trainee doctors with at least one month of working experience; and (ii) undergoing training in all clinical departments (i.e., those who are actively involved in clinical work). However, those who were on maternity or medical leaves during the recruitment period were excluded from the study. All trainee doctors receive monthly wages from the Ministry of Health, Malaysia.

### 2.4 Sample size estimation

The sample size for the study was calculated using Epi Info (version 7.2.5). Considering a WPB prevalence rate of 13.3 % based on previous local study [7], a confidence level of 95 %, and a margin of error of 5 %, the estimated sample size necessary for the study was 177 participants. To account for missing data, we inflated the sample size by 40 %, making the final sample size of 295 participants.

### 2.5 Sampling technique

A proportionate stratified sampling method was applied to establish the number of participants required from each of the six hospitals. Then, the researchers obtained a list of names, referred to as the sampling frame, which includes all trainee doctors working in these hospitals. The participants from each hospital were sampled using a simple random technique.

### 2.6 Study procedure

The principal investigator, with the assistance of several site investigators (i.e., medical officers and nurses from the public health unit of each of the six included hospitals) contacted the selected trainee doctors via their personal mobile numbers. Then, questionnaire booklets were distributed to these chosen trainee doctors after their weekly compulsory continuous medical education (CME) sessions which were conducted at the hospitals’ auditoriums.

### 2.7 Study instruments

Four self-administered questionnaires (in English language) combined in a single booklet were distributed to the eligible participants. These included the sociodemographic characteristics questionnaire, job characteristics questionnaire, WPB questionnaire, and the Big Five Inventory-10 (BFI-10). The sociodemographic characteristics questionnaire inquired about the participants’ age, gender, ethnicity, marital status, and educational background. The educational background indicates the institution from which the individual graduated from, which was further classified as local public college, local private college, and overseas college from Australasia, Europe, or the Middle East. The job characteristics questionnaires included items on working experience (in years) and working hours per week (in hours).

The WPB questionnaire was adapted from Ruth et al. [13] to the present study to capture the types of WPB faced by the participants as well as the perpetrators' characteristics. First of all, the participants were asked if they have experienced any form of WPB at their workplace since the beginning of their employment as trainee doctors. Participant will be required to answer “Yes” or “No” honestly based on their working experience and encounter. Participants will be considered as victim of WPB if they answer “Yes” to this question and vice versa, thereby assigning them as having or not having WPB. If they reported “Yes” to this question, they were required to indicate the types of WPB experienced (e.g., verbal abuse, mental abuse, physical bullying, and sexual harassment). Subsequently, the perpetrators' characteristics (e.g., staff category, gender, and department of perpetrators) was also inquired. The participants may choose more than one response for the types of WPB and the perpetrators' characteristics. The WPB questionnaire was originally developed to measure WPB among nurses in Malaysia. The Cronbach's alpha was 0.87, indicating good reliability. To ensure that this questionnaire is of suitable use among the trainee doctors in Malaysia, three trainee doctors were invited to assess the face validity of this questionnaire. The questionnaire was found to be relevant and comprehensive to measure all construct of WPB.

The Big Five Inventory-10 (BFI-10) [14] was utilised to measure the personality traits among the participants. It consists of 10 items distributed over five subscales, namely the extraversion, agreeableness, conscientiousness, neuroticism, and openness. Extraversion pertains to the level at which someone exhibits energy, sociability, chattiness, and sociability. Agreeableness encompasses the extent to which an individual is kind, nurturing, helpful, and cooperative, and is able to maintain positive relationships with others. Conscientiousness involves the degree to which a person is structured, reliable, punctual, goal-oriented, and can be relied upon. Neuroticism indicates the level of worry, anxiety, impulsiveness, and insecurity an individual experience. Openness reflects the extent to which someone possesses a vivid imagination, creativity, curiosity, and an open-minded perspective [15,16]. Participants were required to answer each item using a five-point Likert scale response, ranging from 1 (“strongly disagree”) to 5 (“strongly agree”). Each personality subscale consists of two questions, one with standard scoring and one with reverse scoring. For a particular subscale, scores of six and below are considered low, while scores greater than six are considered high.

### 2.8 Statistical analysis

Statistical analysis was performed with Statistical Package for the Social Sciences (SPSS) version 26. Categorical variables (e.g., sociodemographic characteristics, job characteristics, WPB, and personality traits) were presented in frequency and percentage. Chi-square tests were used to examine the association between: (i) sociodemographic characteristics and WPB, (ii) job characteristics and WPB; and (iii) personality traits and WPB.

In Malaysia, the training period for medical graduates typically spans two years. However, based on our understanding, many trainee doctors fail to meet the requirements of their training programs within this stipulated timeframe due to various reasons, such as competence issues, poor conduct, extended leaves, involvement in serious medical errors, legal complications, or failure in their exit exams. Those trainees who cannot complete their training within the allotted two years, often referred to as “extended trainees”, are frequently labelled as lacking the necessary skills. Consequently, in the Chi-square test, we categorised our study participants based on a two-year cut-off to investigate whether trainee doctors who have worked as trainee doctors for longer than two years are more susceptible to experiencing WPB.

In numerous regions, including Malaysia, medical students typically complete their undergraduate medical programs and graduate between the ages of 24–26. As such, these medical graduates often commence their training at around 25–27 years old and are typically anticipated to finish this training before they turn 30. It is considered atypical for trainee doctors to remain in such junior roles beyond the age of 30. Such cases are often perceived as a sign of incompetence and might be associated with higher level of WPB. This explains the justification of grouping the participants using the cut-off point of 30 years old in the Chi-square test.

Multivariate logistic regression was applied to evaluate the association between the significant independent variables (as determined from chi-square tests) and WPB. Besides that, multiple logistic regression was used to quantify the magnitude of the effect of each significant independent variable on WPB (i.e., which variable is the strongest predictor of WPB) as well as to quantify the simultaneous effects of multiple significant independent variables on WPB. Independent variables that have p-values <0.25 (based on previous chi-square tests) were entered to the stepwise multiple logistic regression model using forward selection criteria.

### 2.9 Ethics consideration

The present study obtained approval from the Medical Research and Ethics Committee (MREC), Ministry of Health, Malaysia (NMRR ID: 21-1633-59237) and the Ethics Committee of the National University of Malaysia (JEP-2021-406) in August 2020. The study participants were informed about the study's objectives and they were assured that the data collected would remain confidential. All participants in the present study were ensured for their anonymity as personal identification was not required on the questionnaire booklet. Additionally, their superiors (e.g., medical officers, specialists, and head of department) were invited to leave the auditoriums throughout the data collection process (i.e., from the distribution of questionnaires to submission of completed questionnaires). Trainee doctors were also reminded that it is their right to refuse to participate to the present study if they feel uncomfortable. Trainee doctors who reported the incidence of WPB will not be identified and notified to the hospital management but were encouraged to report such incidence to the hospital management anonymously. Informed consent was obtained from the participants prior to the study.

## 3 Results

A total of 279 participants (out of 295 participants required) responded to our questionnaires, resulted in a response rate of 94.6 %. Fifteen responses were excluded due to missing data. A final 264 responses were analysed. Among these 264 participants, a large proportion were female (n = 175, 66.3 %), under 30 years old (n = 255, 96.6 %), Malays (n = 159, 60.2 %), single (n = 188, 71.2 %), and graduated from local private universities (n = 110, 41.7 %). In terms of job characteristics, a majority of the participants had working experience of 1–2 years (n = 177, 67.0 %) and worked more than 80 h per week (n = 90, 34.1 %) (Table 1). The age, working experience, and working hours were not normally distributed as tested by the p-value for Kolmogorov-Smirnov test and Q-Q plot.

**Table 1:** Sociodemographic and job characteristics of the participants (N = 264).

Sociodemographic Characteristics	Median (IQR)	Frequency, n	Percentage, %
**Age (years)**	26.8 (26.6–27.1)		
≤30		255	96.6
>30		9	3.4
**Gender**	–		
Female		175	66.3
Male		89	33.7
**Ethnicity**	–		
Malay		159	60.2
Chinese		56	21.2
Indian		45	17.0
Others		4	1.6
**Marital Status**	–		
Married		76	28.8
Single		188	71.2
**Educational Background**	–		
Local public college		77	29.2
Local private college		110	41.7
Overseas college (Australasia)		23	8.7
Overseas college (Europe)		25	9.6
Overseas college (Middle East)		29	11.0
**Working Experience (years)**	2.1 (1.8–2.5)		
Less than 1		73	27.7
1–2		177	67.0
More than 2		14	5.3
**Working Hours Per Week (hours)**	63.7 (55.9–73.6)		
40 - 50		5	1.9
51 - 60		34	12.9
61 - 70		74	28.0
71–80		61	23.1
More than 80		90	34.1

The prevalence of WPB among the participants was 45.1 %. The most common forms of bullying were verbal abuse (n = 193, 46.2 %), followed by mental abuse (n = 183, 43.8 %) and physical violence (n = 28, 6.7 %). A small proportion of the participants were subjected to sexual harassment (n = 14, 3.3 %) (Table 2). The most common perpetrators were found among the professional group, specifically the medical officers (n = 187, 52.4 %) and professional females (n = 149, 53.0 %). Meanwhile, staff nurses (n = 162, 82.2 %) and the females (n = 170, 81.3 %) were the most common perpetrators in the supporting group. Among the non-staff, the most common perpetrators were patients (n = 38, 56.7 %) and the females (n = 47, 53.4 %) (Table 2). In terms of departments, general surgery had the highest prevalence of WPB (n = 144, 25.9 %), followed by obstetrics and gynaecology (n = 118, 21.2 %), general medicine (n = 95, 17.1 %), and orthopaedics (n = 88, 15.9 %) (Table 2).

**Table 2:** Types of WPB and characteristics of the perpetrator.

Variables	Frequency, n	Percentage, %
Types of Violence^a^		
Verbal abuse	193	46.2
Mental abuse	183	43.8
Physical violence	28	6.7
Sexual harassment	14	3.3
**Characteristics of the Perpetrators**^a^		
Staff Category		
**Professional (n = 357)**		
Hospital management	26	7.3
Specialist doctors	75	21.0
Medical officers	187	52.4
Trainee doctors	69	19.3
**Supporting staff (n = 197)**		
Staff nurses	162	82.2
Medical assistants	16	8.2
Other support staffs	19	9.6
**Non-staff (n = 67)**		
Patients	38	56.7
Patient's family members	21	31.3
Visitors	4	6.0
Others	4	6.0
Gender of Perpetrators		
**Professional (n = 281)**		
Males	132	47.0
Females	149	53.0
**Supporting staff (n = 209)**		
Males	39	18.7
Females	170	81.3
**Non-staff (n = 88)**		
Males	41	46.6
Females	47	53.4
Department of Perpetrators		
General medicine	95	17.1
General surgery	144	25.9
Obstetrics and gynaecology	118	21.2
Paediatrics	51	9.2
Orthopaedics	88	15.8
Emergency medicine	21	3.7
Anaesthesiology	35	6.2
Psychiatric	2	0.4
Primary care	3	0.5

a Participants can choose more than one answer.

Chi-square tests showed a significant association between marital status and WPB (X^2^ = 5.70, p = 0.03). This is reported in Table 3. Other socioeconomic factors and all job characteristics were not associated with WPB. In terms of personality traits, only agreeableness (X^2^ = 8.12, p = 0.01) and conscientiousness (X^2^ = 4.24, p = 0.04) had significant associations with WPB (Table 4). Other personality traits such as extraversion, conscientiousness, neuroticism, and openness did not predict WPB.

**Table 3:** The association between sociodemographic and job characteristics with workplace bullying.

	Workplace Bullying			
Risk factors	Yes (n%)	No (n%)	X^2^	P-value
**Age (years)**			0.97	0.33
≤30	113 (44.3)	142 (55.7)		
>30	6 (66.7)	3 (33.3)		
**Gender**			0.01	0.95
Males	40 (44.9)	49 (55.1)		
Females	79 (45.1)	96 (54.9)		
**Marital Status**			5.70	0.03^b^
Single	76 (40.4)	112 (59.6)		
Married	43 (56.6)	33 (43.4)		
**Ethnicity**^a^			0.01	0.93
Malay	72 (45.3)	87 (54.7)		
Non-Malay	47 (44.8)	58 (55.2)		
**Educational Background**^a^			0.80	0.37
Local institutions	81 (43.3)	106 (56.7)		
Overseas institutions	38 (49.4)	39 (50.6)		
**Working Experience**^a^			0.87	0.35
0–2 years	111 (44.4)	139 (55.6)		
More than 2 years	6 (42.9)	8 (57.1)		
**Working Hours Per Week**^a^**(hours)**			0.14	0.71
≤80	77 (44.3)	97 (55.7)		
>80	42 (46.7)	48 (53.3)		

a Adjustments were made to the ethnic group, educational background, working experience, and working hours. For ethnicity, participants were reclassified into two categories, namely “Malay” and “Non-Malay”. For educational background, participants were reclassified into “Local institutions” and “Overseas institutions”. For working experience, participants were reclassified into “0–2 years” and “more than 2 years”. For working hours per week, participants were reclassified into “≤ 80 h” and “> 80 h”.

b Chi-Square test significant p < 0.05.

**Table 4:** The association between personality characteristics with workplace bullying.

		Workplace Bullying			
Personality Traits	Scores	Yes (n%)	No (n%)	X^2^	p-value
Extraversion				0.20	0.65
	Low	73 (46.2)	85 (53.8)		
	High	46 (43.4)	60 (56.6)		
Agreeableness				8.12	0.01*
	Low	30 (63.8)	17 (36.2)		
	High	89 (41.0)	128 (59.0)		
Conscientiousness				4.24	0.04*
	Low	35 (56.5)	27 (43.5)		
	High	84 (41.6)	118 (58.4)		
Neuroticism				0.14	0.71
	Low	67 (44.1)	85 (55.9)		
	High	52 (46.4)	60 (53.6)		
Openness				0.20	0.66
	Low	77 (46.1)	90 (53.9)		
	High	42 (43.3)	55 (56.7)		

*Chi-Square test significant p < 0.05.

A multivariate logistic regression showed that marital status (p = 0.03) and agreeableness personality (p = 0.02) appeared as significant predictors of WPB among the participants (Table 5). Participants who were married (OR: 1.87; 95 % CI: 1.08–3.23) had higher odds of experiencing WPB than those who were single. In addition, participants with low agreeableness (OR: 2.29; 95 % CI: 1.17–4.47) had higher odds of being bullied at work compared to those with high agreeableness. Conscientiousness did not predict WPB.

**Table 5:** Multivariate logistic regressions to determine the predictors of WPB.

Predictors	P-Value	OR	95 % Confidence Interval	
**Marital Status**				
Single (reference)	0.03*	1.87	1.08	3.23
Married				
**Agreeableness**				
High agreeableness (reference)	0.02*	2.29	1.17	4.47
Low agreeableness				
**Conscientiousness**				
High conscientiousness (reference)	0.13	1.59	0.88	2.89
Low conscientiousness				

*Multivariable logistic regression significant p < 0.05.

## 4 Discussion

The present study found that 45.1 % of the trainee doctors in Selangor, Malaysia experienced WPB. This prevalence is higher compared to another local study, which reported a prevalence of 13.3 % [7]. This higher prevalence could be explained by the fact that the study population in our study were new batches of trainee doctors who were employed under contract basis by the Ministry of Health, Malaysia. In addition, the current study was conducted during the COVID-19 pandemic, a time when workload and working hours of these trainee doctors were extended tremendously, which subsequently translated to higher occupational stress [17,18]. These factors may expose trainee doctors to higher WPB.

Consistent with prior research conducted by Gutierez et al. [19], Surimi et al. [20], and Afolarami et al. [21], a majority of the trainee doctors in our study experienced verbal abuse. Verbal abuse involves the use of words to induce fear and exert control over someone, including actions like yelling, threats, insults, and offensive language [22]. Medical officers were identified as the most frequent perpetrators of WPB against trainee doctors within the professional group, which aligns with findings from earlier studies [7,20]. This type of bullying, known as “downward bullying”, stems from power imbalances between the two parties (i.e., trainee doctors and medical officers) [23]. Furthermore, within the support staff group, nurses were found to be more likely to engage in WPB towards trainee doctors. This form of bullying, occurring between colleagues at a similar power level, is referred to as “horizontal bullying” [23]. Additionally, instances of bullying by non-staff, such as patients and their family members, could be influenced by factors such as insufficient time dedicated to patients, inadequate doctor-patient communication, long waiting times, overcrowding in waiting areas, lack of trust in trainee doctors, and dissatisfaction with treatment or care provided [24]. With regards to the departments where trainee doctors worked, WPB was most prevalent in general surgery and other surgical-based departments, corroborating findings from previous studies [19]. This could be attributed to the high stress levels, demanding workloads, and increased risk of medico-legal consequences associated with these departments [25], which heightens the potential for workplace aggression and bullying.

It is also important to highlight that there was an association between marital status and the level of WPB, in which married trainee doctors experienced almost double the odds of WPB compared to single trainee doctors. Although marriage itself does not directly cause WPB, there can be indirect and complex ways in which marriage can influence workplace dynamics. For instance, challenges in maintaining a healthy work-life balance, including balancing the demands of marriage or family life with work responsibilities, can impact an individual's stress levels and overall psychological well-being. This imbalance might indirectly affect an individual's susceptibility to WPB [26]. Besides this, marital stress can alter morbidity and mortality risks via a neuroimmunological mechanism. When an individual's physical health is compromised, it can have a ripple effect on their professional life, influencing their work productivity, ability to focus, and interactions with co-workers. This may make them more susceptible to experiencing WPB [27].

Of note, low agreeableness was also a predictor of WPB, as previously observed in a study conducted in Sweden [28]. Low agreeableness is characterised by traits such as skepticism, challenging others' ideas, unwillingness to engage, self-centeredness, jealousy, cynicism, rudeness, suspicion, uncooperativeness, ruthlessness, irritability, and manipulation. It also involves a focus on one's own goals and interests while lacking sympathy for others. Consequently, trainee doctors with low agreeableness tend to have poor interpersonal relationships with their colleagues, thereby increasing their vulnerability to WPB [29].

It is surprising that extraversion, conscientiousness, and neuroticism were not associated with WPB in the present study. This finding could be possibly explained by the presence of social desirability bias [30], in which trainee doctors might provide inaccurate responses that they believe will impress the researchers (i.e., by showing high extraversion and conscientious) and at the same time concealing their weaknesses (e.g., by hiding their high neuroticism). On the other hand, while openness is proven to be associated with WPB in other industrial sectors [31], the healthcare sector rarely emphasise on this particular personality. In fact, trainee doctors are only required to follow instructions given by their superiors as well as doing “the right thing at the right time”, but there is no need for trainee doctors to be “creative, curious, and explore new ideas”. This explains why such association was not found in the present study.

The findings in our study underscores the importance of implementing effective strategies to reduce WPB to safeguard the physical and mental well-being of trainee doctors. As such, we would like to propose a few strategies to address WPB based on a Cochrane review [32]. Firstly, all health care institutions should adopt a “zero-tolerance” policy towards WPB. Hospital management needs to foster a supportive culture and emphasise to all staff members that all forms of WPB are considered serious offenses. Superiors must handle reports of WPB with utmost seriousness and whistleblowers should be protected. Efforts should also be made to reduce workplace stressors that contribute to WPB. Furthermore, additional support in the form of stress management training shall be offered to all trainee doctors, especially for those who are married. Trainee doctors identified with low agreeableness should be given cognitive behavioural therapy.

## 5 Limitations

The present study had a few limitations that need to be acknowledged. Firstly, it is important to recognise that scolding and reprimanding by senior officers can occur frequently in any health care facility. While these actions are often deemed as necessary to address any wrongdoing and poor behaviours among trainee doctors, ensuring patient safety and promoting proper learning, trainee doctors may interpret these remedial actions as abusive or bullying. This perception could introduce bias into their responses. Secondly, since participants were asked to recall instances of bullying since the beginning of their employment, there is a possibility of inaccuracies in reporting the number of WPB and identifying the exact perpetrators, which could lead to recall bias. Thirdly, this study only included trainee doctors who are currently employed, potentially overlooking individuals who have resigned from the healthcare system due to serious impacts of WPB.

Besides these, very often trainee doctors are ridiculed, verbally degraded, and demotivated to continue training by their superiors by pointing out their lower intelligence and/or poor practical learning skills. Unfortunately, these cannot be captured in the Big Five Inventory-10 as this tool is designed to measure personality only. Finally, we acknowledge the fact that there were changes in the infection status of COVID-19 throughout the study period. In fact, according to WHO, there were two surges of cases in mid-August 2021 and at end of February 2022 [33]. Hence, we are unsure how such changes in patient-load and workload influence the personality traits of the trainee workers, which in turn affect the prevalence of WPB during the COVID-19 pandemic.

## 6 Conclusion

The high prevalence of WPB among trainee doctors could be attributed by marriage and low agreeableness personality traits in this population. In order to minimise WPB and maximise workforce potential, it is essential for healthcare institutions and medical training programmes to recognise these vulnerabilities and take steps to protect and support trainee doctors who are married and/or with low agreeableness personality trait throughout their professional career development.

## Funding information

None.

## Declaration

The present study obtained approval from the Medical Research and Ethics Committee (MREC), Ministry of Health, Malaysia (NMRR ID: 21-1633-59237) and the Ethics Committee of the National University of Malaysia (JEP-2021-406) in August 2020. All study participants provided informed consent prior to their participations to the present study. The authors declare that there is no conflict of interest.

## Data availability statement

Data pertaining to the present study has not been deposited into a publicly available repository. However, it will be made available upon request.

## CRediT authorship contribution statement

**Halim Ismail:** Supervision, Methodology, Conceptualization. **Dzualkamal Dawam:** Formal analysis, Data curation. **Nor Azila Muhd Aris:** Formal analysis, Data curation. **Sheng Qian Yew:** Writing – review & editing, Writing – original draft, Supervision, Formal analysis. **Hanis Ahmad:** Formal analysis, Data curation. **Chan Chee Hoong David:** Formal analysis, Data curation. **Mohd Hafiz Baharudin:** Formal analysis, Data curation. **Zhe Shen Huam:** Formal analysis, Data curation. **Hibatul Hakimi Jamaludin:** Formal analysis, Data curation.

## Declaration of competing interest

The authors declare that they have no known competing financial interests or personal relationships that could have appeared to influence the work reported in this paper.
