# Supplementary material for: Personality traits and workplace bullying among contract trainee doctors in Malaysia
Source: Heliyon. 2023 Dec 12;10(1):e23625. doi: 10.1016/j.heliyon.2023.e23625 (PMC10761799; doi:10.1016/j.heliyon.2023.e23625)
Supplement: Multimedia component 1 [file mmc1.docx]

**Supplementary Material**

**Section A: Sociodemographic Characteristics**

**Kindly select ONE answer only unless stated otherwise**

A1 Age: (_____ years old)

A2 Gender:

- 1 Male
- 2 Female

A3 Ethnicity:

- 1 Malay
- 2 Chinese
- 3 Indian
- 4 Others (_________________)

A4 Marital status:

- 1 Married
- 2 Single (including divorced)

A5 Received medical education from:

- 1 Local public college
- 2 Local private college
- 3 Overseas college (Australasia)
- 4 Overseas college (Europe)
- 5 Overseas college (Middle East)

**Section B: Job Characteristics**

**Kindly select ONE answer only unless stated otherwise**

B1 Working experience as houseman:

- 1 Less than 1 year
- 2 1-2 years
- 3 More than 2 years

B2 How long do you work per week?

- 1 40-50 hours
- 2 51-60 hours
- 3 61-70 hours
- 4 71-80 hours
- 5 More than 80 hours

**Section C: Workplace Bullying Questionnaire**

**Kindly select ONE answer only unless stated otherwise**

C1 Have you ever been bullied at work since you started housemanship?

- 1 Yes
- 2 No

C2 If you answer “Yes” to the above question, what is/are the type of bullying you ever experienced? (you may select more than one answer)

- 1 Verbal abuse
- 2 Mental abuse
- 3 Physical violence
- 4 Sexual harassment

C3 Who of the following have bullied you at work? (you may select more than one answer)

- 1 Hospital management (Professional)
- 2 Specialist doctors (Professional)
- 3 Medical officers (Professional)
- 4 Trainee doctors (Professional)
- 5 Staff nurses (Supporting staff)
- 6 Medical assistants (Supporting staff)
- 7 Other supporting staff (Supporting staff)
- 8 Patients (Non-staff)
- 9 Patients’ family members (Non-staff)
- 10 Visitors (Non-Staff)
- 11 Others (______________________)

C4 Genders of the perpetrators (the one who most frequently or severely bully you at work):

Professional

- 1 Male
- 2 Female

Supporting staff

- 1 Male
- 2 Female

Non-staff

- 1 Male
- 2 Female

C5 At which department have you been bullied? (you may select more than one answer)

- 1 General medicine
- 2 General surgery
- 3 Obstetrics and Gynaecology
- 4 Paediatrics
- 5 Orthopaedics
- 6 Emergency medicine
- 7 Anaesthesiology
- 8 Psychiatric
- 9 Primary care

**Section D: Big Five Inventory (BFI-10)**

**Kindly select ONE response that best describe your personality for each of the following statement**

| **I see myself as someone who** | **Disagree strongly** | **Disagree a little** | **Neither agree nor disagree** | **Agree a little** | **Agree strongly** |
| --- | --- | --- | --- | --- | --- |
| 1. Is reserved | 1 | 2 | 3 | 4 | 5 |
| 1. Is generally trusting | 1 | 2 | 3 | 4 | 5 |
| 1. Tends to be lazy | 1 | 2 | 3 | 4 | 5 |
| 1. Is relaxed, handles stress well | 1 | 2 | 3 | 4 | 5 |
| 1. Has few artistic interests | 1 | 2 | 3 | 4 | 5 |
| 1. Is outgoing, sociable | 1 | 2 | 3 | 4 | 5 |
| 1. Tends to find fault with others | 1 | 2 | 3 | 4 | 5 |
| 1. Does a thorough job | 1 | 2 | 3 | 4 | 5 |
| 1. Gets nervous easily | 1 | 2 | 3 | 4 | 5 |
| 1. Has an active imagination | 1 | 2 | 3 | 4 | 5 |
